# Locational and Directional Dependencies of Smooth Muscle Properties in Pig Urinary Bladder

**DOI:** 10.3389/fphys.2019.00063

**Published:** 2019-02-06

**Authors:** Mischa Borsdorf, André Tomalka, Norman Stutzig, Enrique Morales-Orcajo, Markus Böl, Tobias Siebert

**Affiliations:** ^1^Institute for Sport and Exercise Science, Department of Movement and Exercise Science, University of Stuttgart, Stuttgart, Germany; ^2^Institute of Solid Mechanics, Department of Mechanical Engineering, Technical University of Braunschweig, Braunschweig, Germany

**Keywords:** urinary bladder wall, contractile properties, regional dependencies, tissue composition, fiber type, lower urinary tract, bladder morphology

## Abstract

The urinary bladder is a distensible hollow muscular organ, which allows huge changes in size during absorption, storage and micturition. Pathological alterations of biomechanical properties can lead to bladder dysfunction and loss in quality of life. To understand and treat bladder diseases, the mechanisms of the healthy urinary bladder need to be determined. Thus, a series of studies focused on the detrusor muscle, a layer of urinary bladder made of smooth muscle fibers arranged in longitudinal and circumferential orientation. However, little is known about whether its active muscle properties differ depending on location and direction. This study aimed to investigate the porcine bladder for heterogeneous (six different locations) and anisotropic (longitudinal vs. circumferential) contractile properties including the force-length-(FLR) and force-velocity-relationship (FVR). Therefore, smooth muscle tissue strips with longitudinal and circumferential direction have been prepared from different bladder locations (apex dorsal, apex ventral, body dorsal, body ventral, trigone dorsal, trigone ventral). FLR and FVR have been determined by a series of isometric and isotonic contractions. Additionally, histological analyses were conducted to determine smooth muscle content and fiber orientation. Mechanical and histological examinations were carried out on 94 and 36 samples, respectively. The results showed that maximum active stress (*p_act_*) of the bladder strips was higher in the longitudinal compared to the circumferential direction. This is in line with our histological investigation showing a higher smooth muscle content in the bladder strips in the longitudinal direction. However, normalization of maximum strip force by the cross-sectional area (CSA) of smooth muscle fibers yielded similar smooth muscle maximum stresses (165.4 ± 29.6 kPa), independent of strip direction. Active muscle properties (FLR, FVR) showed no locational differences. The trigone exhibited higher passive stress (*p_pass_*) than the body. Moreover, the bladder exhibited greater *p_pass_* in the longitudinal than circumferential direction which might be attributed to its microstructure (more longitudinal arrangement of muscle fibers). This study provides a valuable dataset for the development of constitutive computational models of the healthy urinary bladder. These models are relevant from a medical standpoint, as they contribute to the basic understanding of the function of the bladder in health and disease.

## Introduction

**FIGURE 1:**
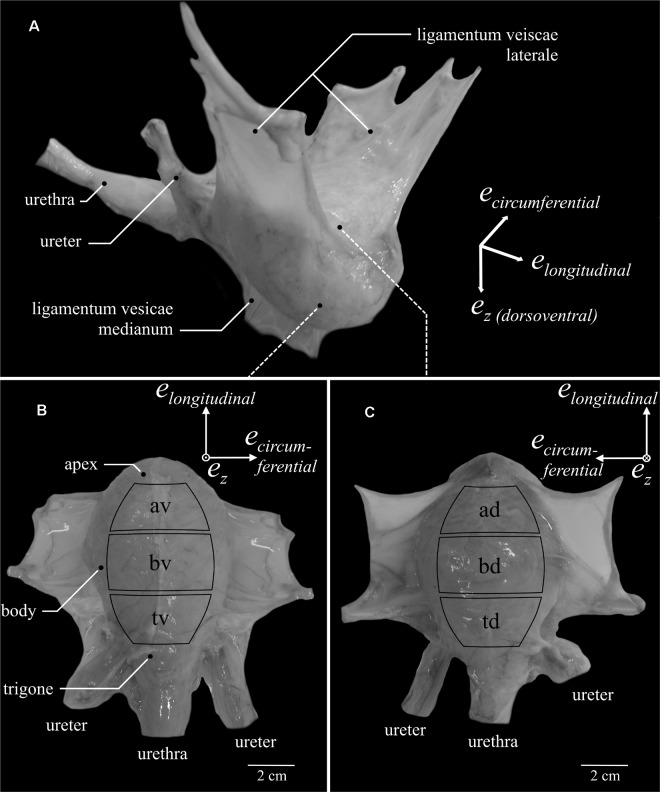
Porcine urinary bladder anatomy and definition of locations. A bladder filled with 300 ml Krebs solution is depicted **(A)** as it is suspended in the female pig. Note, only one ureter can be seen. **(B)** Ventral (av – apex ventral, bv – body ventral, tv – trigone ventral) and **(C)** dorsal (ad – apex dorsal, bd – body dorsal, td – trigone dorsal) views of the bladder with extended *ligamenta vesicae lateralia*. Curved black rectangles indicate the six locations from which tissue samples were dissected.

The urinary bladder is a hollow organ, which absorbs, stores and releases urine, and is thereby exposed to enormous deformation. The bladder (Figure 1A) is located at the base of the pelvic floor superior to the reproductive organs and ventral to the rectum. The bladder can be divided into three anatomical regions (from cranial to caudal): the apex (dome), the body (central region) and the trigone (base) (Figure 1B). The bladder wall consists of several layers (from the inside out): tunica mucosa, tunica submucosa, tunica muscularis (*M. detrusor vesicae*), and tunica serosa. The detrusor consists of sub-layers of smooth muscle cells, which are predominantly arranged in longitudinal and circumferential orientation (Morales-Orcajo et al., 2018). The detrusor muscle is of special physiological and medical interest, as it provides contraction during micturition. It generates high inner pressure by a coordinated, fast reduction of bladder volume in order to successfully propel urine out of the body. The required muscle forces mainly depend on biomechanical properties such as the force-length- (FLR) and the force-velocity-relationship (FVR). Characterization of these biomechanical properties is essential to better understanding bladder functioning. Age (Chantereau et al., 2014; Cheng et al., 2018), disease (Wang et al., 2009) or injuries (Gloeckner et al., 2002; Nagatomi et al., 2004) can alter the biomechanical properties of the bladder wall, resulting in certain bladder dysfunctions. The associated lower urinary tract symptoms represent a global health problem (Irwin et al., 2006), whereby common methods of treatment such as bladder augmentation surgery or transurethral resection of the prostate have short- and long-term complications (Madersbacher and Marberger, 1999; Greenwell et al., 2001; Gerharz et al., 2003; Ahyai et al., 2010). The specific understanding of healthy urinary bladder functioning is a basic prerequisite to identify the causes of disease and to advance medical treatment. Realistic three-dimensional models can describe the function of whole organs in health and disease (Cheng et al., 2007, 2010). Nonetheless, the development of a computational model requires the determination of characteristic passive and active biomechanical and physiological properties (FLR and FVR). For such investigations, whole bladder experiments are insufficient and *in vitro* tests on isolated tissue strips are required. These, in turn, are hardly possible to conduct on intact human bladder tissue. Alternatively, the examination of pig bladder strips is of special importance due to the structural and mechanical similarities shared with the human bladder (Teufl et al., 1997; Dahms et al., 1998).

Most studies on active and passive bladder property determination have used single tissue strips removed from only one specific bladder region, with a specific strip direction (longitudinal or circumferential) (Uvelius, 1976, 1977, 1979, 2001; Griffiths et al., 1979; van Mastrigt, 1988; Longhurst et al., 1990; Minekus and van Mastrigt, 2001; Speich et al., 2007; Menzel et al., 2017; Seydewitz et al., 2017). Therefore, it is an important question whether differences in tissue strip properties exist depending on location (heterogeneity) and/or on tissue strip direction (anisotropy).

Porcine urinary bladders were found to display anisotropic (Gilbert et al., 2008; Korossis et al., 2009; Zanetti et al., 2012; Natali et al., 2015; Seydewitz et al., 2017; Morales-Orcajo et al., 2018) and heterogeneous (Korossis et al., 2009; Morales-Orcajo et al., 2018) passive behavior. The trigone showed higher passive stress than the body and the apex (Korossis et al., 2009; Morales-Orcajo et al., 2018). Results on whether longitudinal or circumferential tissue direction exhibit higher passive stiffness were ambivalent (Gilbert et al., 2008; Korossis et al., 2009; Zanetti et al., 2012; Natali et al., 2015; Seydewitz et al., 2017; Morales-Orcajo et al., 2018). Histological investigations demonstrated regional microstructural differences concerning bladder wall composition (Korossis et al., 2009; Morales-Orcajo et al., 2018) and smooth muscle fiber orientation (Morales-Orcajo et al., 2018). It has been reported that the portion of the detrusor was decreased in the trigone and that smooth muscle fiber orientation was anisotropic on the ventral side of the bladder (Morales-Orcajo et al., 2018). Consequently, it might be assumed that the active properties vary accordingly. An elaborate investigation of contractile properties accompanied by histological analyses might reveal locational or directional dependencies and enable us to draw conclusions about the underlying force-generating mechanisms. In contrast to the series of studies examining location- and direction-dependent passive bladder properties, only a limited number of studies examining active muscle properties exist. Seydewitz et al. (2017) reported differences in maximum stress between longitudinally and circumferentially directed tissue strips taken from one bladder location (dorsal apex) only. Comparing tissue strips isolated from the ventral and dorsal side of the bladder, Longhurst et al. (1995) found small differences in the contractile responsiveness of the strips but no differences between their force-length relations. Therefore, a systematic investigation of location- and direction-dependent active muscle properties is required for the development of more integrative and realistic urinary bladder models.

Therefore, the present study is the first to perform *in vitro* experiments on porcine bladder tissue strips, determining active biomechanical properties (such as FLR and FVR) of six distinct urinary bladder locations through uniaxial tests in both longitudinal and circumferential directions. Furthermore, passive characteristics were investigated in specific ramp-length experiments. Additionally, histological analyses were carried out to characterize the smooth muscle content, considering both locational and directional effects. To facilitate comparisons with a recent study on microstructural and passive mechanical differences of pig urinary bladder (Morales-Orcajo et al., 2018), we similarly distinguished between apex, body, and trigone, and the ventral (Figure 1B) and dorsal bladder (Figure 1C).

## Materials and Methods

### Experimental Set-Up

The study was exempted from ethical committee review according to national regulations (German Animal Welfare Act), as urinary bladders of healthy, female domestic pigs (*Sus scrofa domestica*, age: ≈6 month, mass: ≈100 kg) were obtained from a slaughterhouse immediately after animal sacrifice. The experiments were conducted on 43 urinary bladders within 10 h after death. The experimental set-up, preparation, and handling of bladder smooth muscle tissue have previously been described in detail (Menzel et al., 2017; Tomalka et al., 2017). An aerated (95% O2 and 5% CO2) Krebs solution (25 mM NaHCO3; 1.2 mM NaH2PO4; 2.4 mM MgSO4; 5.9 mM KCL; 2.5 mM CaCl2; 117 mM NaCl and 11 mM C6H12O6; pH 7.4) (van Mastrigt and Glerum, 1985) was used for storage of the tissue and realization of the experiments at temperatures of 4 and 37°C, respectively. Similar to a recent study (Morales-Orcajo et al., 2018), the bladder was categorized into three regions (apex, body, and trigone) and two sides (ventral and dorsal), resulting in six locations (Figure 1B,C, apex dorsal [ad], apex ventral [av], body dorsal [bd], body ventral [bv], trigone dorsal [td], trigone ventral [tv]). The entire bladders were dissected along the *ligamentum vesicae medianum*. Subsequently, tissue strips (0.93 ± 0.24 g) of 16 × 8 mm were cut out of the designated locations (Figure 1B,C) either in the longitudinal (along the apex to trigone line) or circumferential direction. The strips were mounted vertically to a motor/force transducer (Aurora Scientific 305C-LR dual mode muscle lever system). The strip was suspended in a relaxed state at a very short length (clearly sagging). Then, the strip was passively lengthened through an isokinetic ramp (5 mm/min) until a passive force of 5–10 mN was reached. After finishing the ramp, the strip was kept at this length, while the passive force development settled at a negligible baseline value (<1 mN). The length of the strip, averaging 12.41 ± 2.08 mm between clamp and attachment hooks, was determined with a digital sliding caliper and was defined as slack length (*L_S_*). Muscle contractions were induced by electrical stimulations (900 mA, 100 Hz, 5 ms) (van Mastrigt and Glerum, 1985) for about 12 s. The muscle properties were determined after an equilibration period of 30 min and as soon as a stable force generation (deviation < 5% of active isometric force) was attained.

### Determination of Muscle Properties

Uniaxial experiments were performed *in vitro* on 94 tissue strips (*n* = 94). Table 1 summarizes the number of samples examined for each analyzed parameter. The protocols used to determine the FLR and FVR have been described earlier (Menzel et al., 2017; Tomalka et al., 2017). Briefly, the FLR was investigated by a series of isometric contractions with increasing muscle length (*L*) until passive forces reached 50% of the maximum isometric force (*F_im_*). The muscle length at *F_im_* was defined as the optimal muscle length (*L*_0_). The ascending and descending limbs of the FLR were fitted using linear regression models (*f*(*x*) = *mx* + *b*, with *x* = *L/L_S_*) to extrapolate to lengths of zero force generation. The respective distances to *L*_0_ were defined as the width of the ascending (*w_asc_*) and descending limb (*w_desc_*). The FVR was determined by isotonic contractions at *L*_0_ against forces of 0.1–0.9 *F_im_*. The data were fitted with Hill’s hyperbolic equation (Hill, 1938) to yield the maximum shortening velocity (*v_max_*) and the curvature factor *curv = a/F_im_* (damping increases with decreasing *curv; a* describes the force asymptote) (Siebert et al., 2017). Between all contractions, recovery phases of 5 min were interposed and the “cycling-protocol” by Brenner (1983) was applied.

**Table 1:** The number of investigated tissue samples of each group for the observed muscle properties and parameters.

				FLR					FVR	
				*L*_0_	*w_asc_*	*w_desc_*	*p_act_*	*p_pass_*	*v_max_*	*curv*
Number of samples	Longitudinal	Trigone	ventral	6					7	
			dorsal	6					7	
		Body	ventral	6					6	
			dorsal	6					6	
		Apex	ventral	7					8	
			dorsal	6					11	
	Circumferential	Trigone	ventral	6					6	
			dorsal	6					6	
		Body	ventral	6					6	
			dorsal	6					6	
		Apex	ventral	6					6	
			dorsal	6					6	

The CSA was calculated by the product of the thickness and the width of the tissue strip at *L*_0_, assuming a rectangular cross-section. The active and passive isometric forces at *L*_0_ were divided by the strip’s CSA to calculate the maximum active stress (*p_act_*) and the corresponding passive stress (*p_pass_*), respectively.

### Histological Observation

Histological examinations were performed in accordance with previous studies (Menzel et al., 2017; Tomalka et al., 2017). In total, *n* = 36 longitudinal and circumferential tissue strips from the designated locations (Figure 1B,C) were cut out of one representative urinary bladder. Subsequently, the strips were stretched to 300% *L_S_* (to be the strip length where *F_im_* is generated) in the corresponding direction and fast frozen in an isopentane bath cooled with liquid nitrogen. A cryomicrotome was used to cut cross-sectional and lengthwise-sectional slices from the frozen samples, which were stained with Picrosirius Red staining protocol (Morales-Orcajo et al., 2018) and photographed using a digital microscope (Zeiss Smartzoom 5). The smooth muscle content was determined through a color threshold using an image editing software (ImageJ 1.49 v, National Institutes of Health, United States). Stretched muscle fibers in strip direction (which were expected to generate force) were clearly recognizable from lengthwise sections.

The thickness of stretched sublayers was measured from lengthwise sections (Figure 5A) and used to determine the amount of lengthwise-oriented muscle bundles in cross-sectional slices (Figure 5B). The area of stretched muscle fibers normalized to the CSA was defined as the force-generating smooth muscle content (*SMC_stretched_*).

### Statistics

For further statistical analyses, data of muscle properties were normalized as follows: (1) Force and length values were divided by individual *F_im_* and *L*_0_, respectively. (2) Velocity data were normalized to optimum muscle length and expressed in *L*_0_/s. For comparison of pooled bladder locations, the strips were assigned according to their particular region to apex, body, or trigone (ABT) and to their position on the ventral or dorsal side of the bladder (VD) (Figure 1B,C). Additionally, the strips were differentiated according to their direction (longitudinal or circumferential). This results in 12 groups (3 ABT × 2 VD × 2 directions). The biomechanical properties were analyzed using seven parameters (*L*_0_*_,_ w_asc_*, *w_desc_*, *p_act_*, *p_pass_*, *v_max_*, and *curv*). Each parameter was investigated using a 3-way ANOVA (DIRECTION [longitudinal vs. circumferential] × VD [ventral vs. dorsal] × ABT [apex vs. body vs. trigone]). Additionally, *SMC_stretched_*, which was observed through 36 strips from one bladder, was tested for significant locational and directional differences using an ANOVA with repeated measures and a paired *t*-test, respectively. The effect sizes were calculated using partial eta squared ($\eta_{p}^{2}$) and classified as low ($\eta_{p}^{2}$ = 0.01), medium ($\eta_{p}^{2}$ = 0.06), and high ($\eta_{p}^{2}$ = 0.14) (Cohen, 1988). Significant main effects or interactions were followed up by a Bonferroni *post hoc* test. A significance level of *P* < 0.05 was used for all analyses. Statistical analyses were carried out using SPSS 23 (IBM Corp., Armonk, NY, United States).

## Results

**FIGURE 2:**
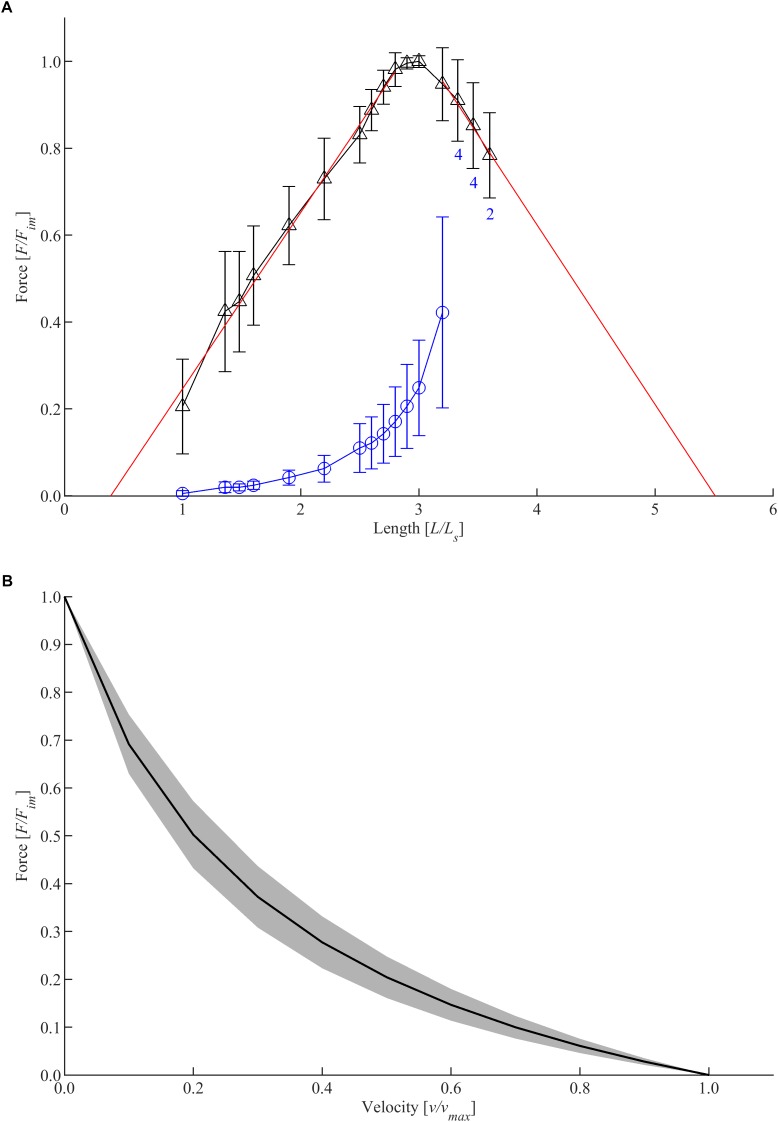
Muscle properties of longitudinal strips dissected from the dorsal body. **(A)** Force-length relationship. Length and force are normalized to slack length (*L_S_*) and maximum isometric force (*F_im_*), respectively. Triangles and circles depict mean values of active and passive isometric force, respectively. Whiskers indicate corresponding standard deviations. Red solid lines represent the ascending (*f*_1_) and descending limb (*f*_2_) of the force-length relationship, which were fitted by linear regression models following the equations: *f*_1_(*x*) = 0.4057*x* − 0.1594 and *f*_2_(*x*) = − 0.4138*x* + 2.279, with *x* = *L*/*L_S_*. Isometric force-length measurements comprise *n* = 6 tissue samples up to lengths of 3.2 *L_S_*; varying sample sizes were investigated at greater lengths (labeled with blue numbers below standard deviation bars on the descending limb). **(B)** Force-velocity relationship. Velocity and force values are normalized to *v_max_* and *F_im_*, respectively. Based on a series of isotonic contractions, force-velocity curves of *n* = 6 tissue samples were fitted using the typical Hill equation (Hill, 1938). The black curve and a gray area indicate mean values and standard deviations, respectively.

Typical FLR and FVR were shown (longitudinal strips from body dorsal) in Figure 2. The shape of the active isometric force-length relationship is similar for all groups investigated. Specifically, the FLR exhibits a linear ascending limb, a narrow bell-shaped plateau region, and a linear descending limb (Figure 2A). Thus, the FLR resembles an inverted parabola. The passive force-length dependency of the bladder tissue strip was characterized by an exponential increase of force with muscle length. The mean *F_im_* of the bladder tissue strips was 1272.86 ± 201.47 mN (*n* = 94). Determining an average CSA of 36.65 ± 6.73 mm^2^ of the whole bladder strips, this corresponds to *p_act_* of 35.16 ± 6.82 kPa. Active force generation occurred at lengths between 0.43 ± 0.41 *L_S_* and 6.66 ± 2.37 *L_S_*, corresponding to 0.138 ± 0.135 *L*_0_ and 2.17 ± 0.74 *L*_0_, respectively. *F_im_* was reached at 3.07 ± 0.28 *L_S_*, accompanied by passive forces of 27.15 ± 9.14% *F_im_*, resulting in a mean *p_pass_* of 9.26 ± 2.71 kPa. The tissue strips had an absolute length of 37.9 ± 4.83 mm at *L*_0_. The total FVR exhibited a typical hyperbolic shape (Figure 2B). The maximum shortening velocity was 2.68 ± 0.69 mm/s, corresponding to 0.071 ± 0.017 *L*_0_*/*s. The mean *curv* was 0.371 ± 0.144.

**FIGURE 3:**
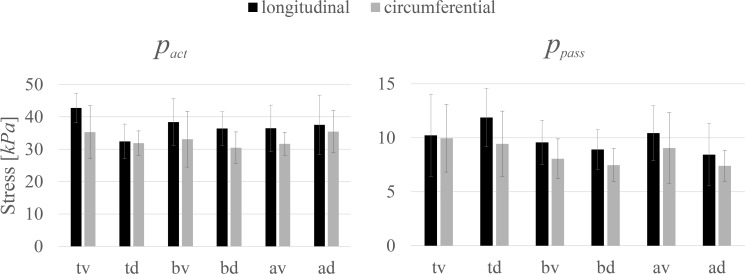
The dependency of maximum active isometric stress (*p_act_*) and passive stress (*p_pass_*) at optimal length (*L*_0_) on location and direction. Mean values ± standard deviations of longitudinal (black) and circumferential (gray) tissue samples are given.

**FIGURE 4:**
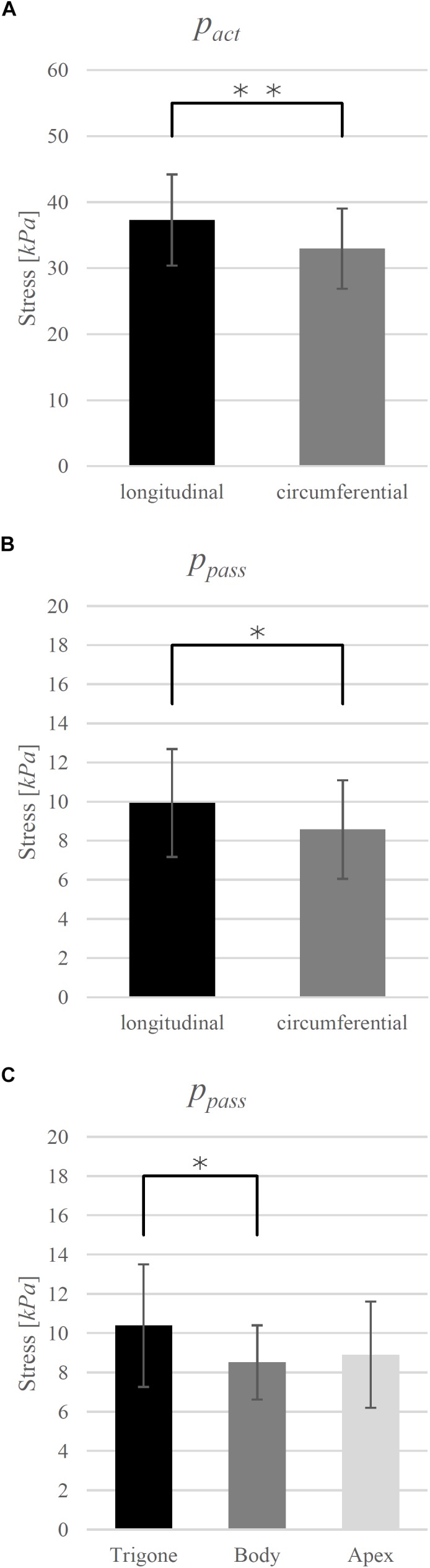
**(A)** Direction dependency of maximum active isometric stress (*p_act_*) and **(B)** passive stress (*p_pass_*) at optimal length (*L*_0_) and **(C)** region-dependency of *p_pass_*. Mean values ± standard deviations are given. Brackets and asterisks (^∗^) mark significant differences in stress. Significance levels are marked as ^∗^*P* < 0.05 and ^∗∗^*P* < 0.01.

The investigated biomechanical parameters for the 12 groups are presented in Table 2. No statistically significant differences were found between *L*_0_, *w_asc_*, *w_desc_*, v*_max_*, and *curv*. Location-specific differences in *p_act_* and *p_pass_* for longitudinally and circumferentially directed bladder strips are summarized in Figure 3. Tissue strips in the longitudinal direction exhibited a significantly higher *p_act_* (*F*_1,61_ = 8.206, *P* = 0.006, $\eta_{p}^{2}$ = 0.119, Figure 4A) and *p_pass_* (*F*_1,61_ = 4.844, *P* = 0.032, $\eta_{p}^{2}$ = 0.074, Figure 4B) than circumferentially directed strips. Furthermore, *p_pass_* differed significantly between regions (*F*_2,61_ = 3.529, *P* = 0.035, $\eta_{p}^{2}$ = 0.104, Figure 4C). Strips from the trigone showed significantly higher *p_pass_* than strips from the body (*P* = 0.047, Figure 4C).

**Table 2:** Location dependency of optimal length (*L*_0_), the width of ascending (*w_asc_*) and descending limb (*w_desc_*) of the force-length relationship, maximum shortening velocity (*v_max_*), and curvature factor (*curv*) of the force-velocity relationship.

Direction	Location	*L*_0_ [*L/L_S_*]	*w_asc_* [*L/L*_0_]	*w_desc_* [*L/L*_0_]	*v_max_* [*L*_0_/s]	*curv*
Longitudinal	tv	3.18 ± 0.36	0.95 ± 0.31	0.98 ± 0.52	0.073 ± 0.023	0.383 ± 0.149
	td	2.96 ± 0.33	0.81 ± 0.12	1.48 ± 1.21	0.066 ± 0.017	0.428 ± 0.155
	bv	3.08 ± 0.26	0.83 ± 0.09	0.90 ± 0.55	0.076 ± 0.019	0.328 ± 0.181
	bd	3.03 ± 0.20	0.89 ± 0.14	0.77 ± 0.16	0.072 ± 0.022	0.360 ± 0.120
	av	3.13 ± 0.37	0.93 ± 0.14	0.97 ± 0.28	0.062 ± 0.004	0.338 ± 0.090
	ad	3.08 ± 0.23	0.94 ± 0.15	1.56 ± 1.33	0.077 ± 0.027	0.341 ± 0.221
Circumferential	tv	3.07 ± 0.29	0.83 ± 0.07	0.99 ± 0.19	0.061 ± 0.007	0.455 ± 0.131
	td	3.22 ± 0.19	0.79 ± 0.07	1.38 ± 1.18	0.069 ± 0.010	0.379 ± 0.102
	bv	3.09 ± 0.31	0.85 ± 0.08	1.03 ± 0.51	0.081 ± 0.019	0.405 ± 0.092
	bd	2.99 ± 0.25	0.82 ± 0.07	1.07 ± 0.38	0.077 ± 0.008	0.293 ± 0.135
	av	3.01 ± 0.24	0.84 ± 0.50	1.60 ± 0.77	0.068 ± 0.017	0.451 ± 0.156
	ad	2.98 ± 0.31	0.86 ± 0.13	1.32 ± 0.72	0.069 ± 0.008	0.314 ± 0.056
	Mean	3.07 ± 0.27	0.86 ± 0.14	1.17 ± 0.74	0.071 ± 0.017	0.371 ± 0.144

**FIGURE 5:**
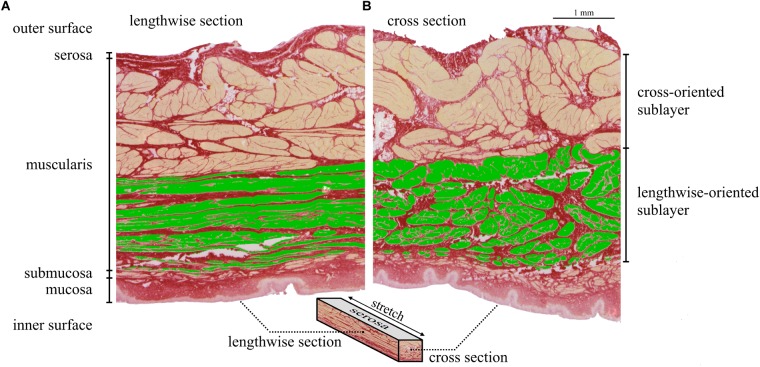
Lengthwise section (**A**: *e_longitudinal_* – *e_z_* plane) and cross-section (**B**: *e_circumferential_* – *e_z_* plane) of a longitudinal strip (td) stretched to 300% *L_S_*. In the lengthwise section **(A)** the lengthwise-oriented stretched muscle fibers are recognizable by their flat, thin shape and marked in green color. The thickness of the lengthwise-oriented sublayer was used to mark the corresponding sublayer in the cross-section **(B)**. Thus, the area of the green colored fibers in the cross-section **(B)** corresponds to lengthwise-oriented muscle fibers, responsible for force generation in strip direction. *SMC_stretched_* is the ratio of the area of lengthwise-oriented muscle fibers and cross-sectional area of the whole strip. Note that the area of stretched muscle fibers (green) appears smaller than that of non-stretched fibers. This is due to a lengthwise stretch of the strip to 300% *L_S_*. In this example (longitudinal strip, td), the lengthwise- and cross-orientated sublayers correspond to sublayers of longitudinal and circumferential smooth muscle fibers, respectively.

Examples of the lengthwise (Figure 5A) and cross-section (Figure 5B) of histologically stained bladder tissues (stretched to 300% *L_S_*) are shown for one tissue strip (longitudinal direction from trigone dorsal). From inside to outside of the bladder wall, the tunica mucosa, submucosa, muscularis, and serosa are distinguishable. Longitudinal strips contained a significantly higher (*P* = 0.015) amount of smooth muscle fibers in the strip direction (*SMC*_stretched_ = 22.34 ± 2.29% CSA) than circumferential strips (*SMC*_stretched_ = 20.63 ± 1.97% CSA) (see Table 3). There were no significant differences in *SMC_stretched_* between regions. Dividing the *p_act_* of longitudinal (37.1 ± 6.91 kPa) and circumferential (33.0 ± 6.08 kPa) strips by their corresponding *SMC_stretched_* yielded no significant differences in active muscle stress (longitudinal 168.6 ± 30.0 kPa; circumferential: 162.2 ± 29.3 kPa; *P* = 0.36). Mean muscle stress of all strips was calculated to be 165.4 ± 29.6 kPa.

**Table 3:** Summary of quantitative histological data.

Strip direction	Region	*SMC_stretched_* [%]	*n*
Longitudinal	Trigone	23.1 ± 2.7	6
	Body	20.7 ± 1.8	6
	Apex	23.3 ± 1.6	6
Circumferential	Trigone	20.5 ± 1.3	6
	Body	20.4 ± 2.6	6
	Apex	21.0 ± 2.1	6

*The force-generating smooth muscle content (SMC_stretched_) is the area of stretched muscle fibers normalized to the CSA. SMC_stretched_ is illustrated exemplarily in Figure 5B (green area).*

## Discussion

To our knowledge, this is the first systematic study investigating active and passive biomechanical as well as histological properties of pig urinary bladder tissue for locational and directional dependencies. Hence, active and passive uniaxial experiments have been performed *in vitro* on whole tissue strips to determine the FLR and FVR at six locations and in two directions.

Although passive characteristics of tissue strips were measured over a large length range (Figure 2), only one parameter, *p_pass_*, was considered for the statistical examination of locational and directional differences in passive behavior. This parameter was determined at *L*_0_ and, thus, was related to *F_im_* and maximal actin-myosin filament overlap. As *L*_0_ did not differ between groups, *p_pass_* is a valid parameter to compare passive stiffness between groups.

To study *SMC_stretched_*, several tissue strips from each location and both directions were histologically examined at *L*_0_. It should be noted that this method categorized smooth muscle content in a dichotomous matter as smooth muscle cells were either aligned to the stretch or in opposition to it, resulting in non-specification of the angle of orientation. Although these strips were excised from only one bladder, the results are in agreement with former histological studies (Morales-Orcajo et al., 2018) and, thus, were suitable for calculation of pure muscle stress and interpretation of mechanical properties. Although biaxial tests can mimic *in vivo* bladder wall deformation in two dimensions, anisotropy can still be adequately detected through uniaxial experiments by measuring in two orthogonal directions.

The urothelium, an epithelial lining at the inner surface of the hollow organs of the urinary tract, consists of approximately 3–5 cell layers corresponding to about 3% of total bladder wall thickness in the rat (Ali et al., 2015). Thus, a negligible effect of the urothelium on passive whole bladder wall properties is expected. However, the urothelium has a modulating effect on smooth muscle contraction and relaxation (Sellers et al., 2018). In the present study, experiments were conducted on whole tissue strips without removal of the urothelium to ensure functional integrity. Several studies using electrical field stimulation of bladder tissue strips reported, that the urothelium inhibited the contractile response (Levin et al., 1995; Chaiyaprasithi et al., 2003, Propping et al., 2013) whereas others found no influence on muscle contraction (Maggi et al., 1987; Kosan et al., 2005). Consequently, the influence of the urothelium on smooth muscle tone should be considered when interpreting force-generating mechanisms. However, as the potential inhibiting effect of the urothelium on smooth muscle contractility was found in multiple bladder regions (Levin et al., 1995), this modulating effect might be negligible when investigating locational and directional differences in biomechanical properties of tissue strips from multiple bladder regions.

An advantage of passive parameters determined within this study, compared to the series of studies (Korossis et al., 2009; Zanetti et al., 2012; Natali et al., 2015) on passive bladder tissue, is that the experiments were conducted on the functionally intact tissue (which was approved by the performance of active muscle contractions over the complete period of experiments) at physiological temperatures of 37°C.

### Detrusor Muscle Properties – Comparison With the Literature

Considering *SMC_stretched_*, the mean maximum stress of the pig detrusor muscle was about 165 kPa and in agreement with maximum stress values typically generated by smooth muscles (Uvelius, 1976; Mashima et al., 1979; Moriya and Miyazaki, 1982; Stephens, 1985; Malmqvist et al., 1991). When dividing strip force by whole strip CSA – as frequently performed in tissue strip studies – the mean *p_act_* (35.2 ± 6.8 kPa) is similar to the stress values observed in previous porcine bladder experiments (van Mastrigt and Glerum, 1985; van Mastrigt, 1988). However, the *p_act_* values in this study are higher compared to a previous study on porcine bladder strips using the same set-up (Menzel et al., 2017). This discrepancy can be explained by the determination of the CSA at *L_S_* (Menzel et al., 2017) in contrast to *L*_0_ (present study). As tissue strip is stretched less at *L_S_* compared to *L*_0_, CSA is larger at *L_S_*, resulting in lower *p_act_* values. However, as *F_im_* is produced at *L*_0_, normalization to *SMC_stretched_* at this length is recommended.

Optimal muscle length *L*_0_ of 3.07 ± 0.28 *L_S_* was similar compared to other studies on the detrusor muscle from pig (Minekus and van Mastrigt, 2001; Menzel et al., 2017; Seydewitz et al., 2017) and other mammals (Longhurst et al., 1990). The extrapolated range at which the muscle produces forces (0.138 to 2.17 *L*_0_) was slightly higher than usually found in smooth muscles (typically ranging within 0.2 to 2 *L*_0_) (Gordon and Siegman, 1971; Mulvany and Warshaw, 1979; Siegman et al., 2013; Tomalka et al., 2017). However, maximal shortenings below 0.2 *L*_0_ have also been reported for rabbit urinary bladder (Uvelius, 1976) or dog tracheal muscle (Stephens et al., 1969). The FLR exhibits a typical bell shape as reported by previous studies on smooth muscles (Herlihy and Murphy, 1973; Mulvany and Warshaw, 1979; Tomalka et al., 2017). Passive forces at *L*_0_ of about 27% *F_im_* comply with previous experiments on smooth muscles (Gordon and Siegman, 1971; Menzel et al., 2017; Tomalka et al., 2017). Mean *curv* factor (0.371 ± 0.144) is within the range of 0.1–0.5 normally found in smooth (Stephens et al., 1969; Gordon and Siegman, 1971; Uvelius, 1977; Moriya and Miyazaki, 1985; Menzel et al., 2017; Tomalka et al., 2017) and skeletal muscles (Siebert et al., 2015). The average *v_max_* of 0.071 ± 0.017 *L*_0_*/*s is within the range of values of 0.04–0.35 *L*_0_*/*s observed in visceral smooth muscles from the pig (Uvelius, 1977; Minekus and van Mastrigt, 2001; Pel et al., 2006; Menzel et al., 2017; Tomalka et al., 2017).

### Active Urinary Bladder Properties – Locational and Directional Dependencies

The detrusor muscle, regardless of location and direction, exhibited a homogeneous shape of the FLR, demonstrating no differences in either *w_asc_*, *w_desc_* or *L*_0_ (Figure 2A). Moreover, the FVR did not differ as *v_max_* and *curv* were similar across all investigated groups (Figure 2B). This is in contrast to the results of Pel et al. (2005), who report differences in *v_max_* (about 20%) and *curv* (about 50%) from muscle fiber bundles, dissected from the outer and inner layer of the porcine detrusor muscle (apex). These differences might be based on the different methods used. In the study of Pel et al. (2005), FVR was determined by a series of isokinetic ramps at different shortening velocities, and velocity values were plotted against force values at the end of the ramp. At this point, the rate of force decrease was still very large, which might strongly influence measurements and, thus, fitting of *v_max_*. Furthermore, FVR data include the force range of 1 to 0.2 *F_im_* corresponding to a velocity range of 0 to 0.5 *v_max_*, which impedes the determination of *v_max_* and *curv*. In contrast, FVR was determined in the present study by performing a series of isotonic and isometric contractions (Siebert et al., 2015; Menzel et al., 2017) covering a larger force range (1 to 0.1 *F_im_*) corresponding to a larger velocity range (0 to 0.7 *v_max_*) and, therefore, allowing a more valid determination of *curv* and *v_max_*.

On the other hand, the *p_act_* values investigated in this study showed dissimilarities, whereby higher values were observed in the longitudinal direction. A recent study analyzed the FLR of isolated detrusor strips from the dorsal apex and also observed higher *p_act_* in longitudinal direction (Seydewitz et al., 2017). These results can be explained by our histological analysis, yielding a higher amount of *SMC_stretched_* in the longitudinal direction compared to the circumferential direction. Normalization of active forces by the CSA of fibers in line with the strip direction yielded no differences of muscle stress between longitudinally and circumferentially oriented smooth muscle fibers. Smooth muscle fibers can be categorized into fast “phasic” or slow “tonic” types (Somlyo and Somlyo, 1968), which differ, among other properties, in contractile kinetics (Horiuti et al., 1989; Gong et al., 1992). The outcomes of this study imply that the detrusor muscle consists predominantly of the “phasic” smooth muscle type, which is in agreement with Boberg et al. (2018), whereby the distribution between “phasic” and “tonic” muscle fibers is uniform across the bladder.

Morales-Orcajo et al. (2018) examined the microstructure of tissue strips at *L_S_* from identical locations of porcine urinary bladder in detail. They observed an inner and outer sublayer of smooth muscle fibers of almost similar thickness and a section in between, showing the high dispersion of orientation. Furthermore, these authors determined a more perpendicular alignment of muscle fibers on the dorsal side, but a more oblique orientation with a longitudinal predominance on the ventral side. A rather longitudinal arrangement of smooth muscle cells is in agreement with our observations of higher *p_pass_*, *p_act,_* and *SMC_stretched_* in the longitudinal compared to circumferential direction (Figure 4). Although the oblique and predominantly longitudinal muscle fiber orientation was observed ventrally (Morales-Orcajo et al., 2018), we did not find any significant differences in the muscle properties between the ventral and dorsal side.

### Passive Urinary Bladder Properties – Locational and Directional Dependencies

We found higher *p_pass_* in the trigone compared to the body (Figure 4C). Collagen is the major contributor for passive stress in the bladder at high stretch levels (Cheng et al., 2018). Several studies examined the relation between passive bladder properties and collagen content and collagen orientation for different bladder regions. Morales-Orcajo et al. (2018) explained higher passive forces in the trigone by increased collagen content. This finding was further supported by Korossis et al. (2009), reporting an increased network of collagen in the trigone.

Our data show higher *p_pass_* in the longitudinal than in circumferential direction (Figure 4B). Some studies observed higher passive stress values in longitudinal (Gilbert et al., 2008; Korossis et al., 2009; Morales-Orcajo et al., 2018), and others in circumferential (Zanetti et al., 2012; Natali et al., 2015; Seydewitz et al., 2017), directions. The corresponding amount and orientation of elastin and collagen were mainly discussed to interpret the findings. Differences in the methodology of previous studies, however, make comparisons rather difficult. A detailed overview of this discrepancy has been made recently (Seydewitz et al., 2017). Morales-Orcajo et al. (2018) have concluded that higher passive stress examined on longitudinal tissue preparations can be explained by the rather longitudinal smooth muscle orientation. Our results of higher *p_pass_* in the longitudinal direction agree with this observation. However, a recent study conducted uniaxial tests on isolated detrusor strips and observed circumferential strips to show greater passive stress values (Seydewitz et al., 2017), performing isokinetic ramps.

### Functional and Physiological Relevance

The detrusor muscle shows no differences in contractile properties across the porcine bladder and might be interpreted as one phasic muscle, which contracts uniformly to void urine over a short period.

At the same time the urethra, which on the other hand has been shown to contain tonic smooth muscle fibers (Hypolite et al., 2001; Pel et al., 2006), relaxes during micturition, and contracts afterward, thereby initiating a new filling process. Unlike the stomach, which autonomously stores, digests, and transports its content, the urinary bladder is (as a complete organ) either in the process of being filled or being emptied. Thus, there seems to be no requirement for functional or locational differences of the detrusor muscle on the whole organ level.

Nonetheless, a predominant longitudinal orientation of smooth muscle cells might not only induce higher stiffness of the bladder along the apex to trigone line but also increase active stress. *In vivo*, the bladder is suspended dorsolaterally through the *ligamenta vesicae lateralia* and ventrally through the *ligamentum vesicae medianum* (Figure 1A). All three ligaments attach along the apex to trigone line. Hence, higher stiffness in the longitudinal direction might reduce their straining during bladder expansion (Morales-Orcajo et al., 2018). Furthermore, as the urethral orifice is located above the bladder in the pig, higher active stress might be required longitudinally to successfully propel urine (against gravitational force) out of the body (Morales-Orcajo et al., 2018). However, it should be noted that due to the upright posture of humans, the bladder position is different, and the urethra is situated on the deepest point of the bladder, facilitating the release of urine. This might lead to differences in bladder microstructure between humans and pigs. Moreover, other external forces, such as pressure exerted by neighboring organs, might influence micturition under physiologic conditions. In addition to the anisotropic and heterogeneous material properties of the bladder, such effects should be integrated into computational whole organ models (Seydewitz et al., 2017) to better understand *in vivo* bladder functioning or to predict functional implications of medical treatments or surgery.

## Conclusion

We examined passive and active properties of bladder tissue strips depending on their location (ad, av, bd, bv, td, tv) and their direction (longitudinal vs. circumferential). Although the bladder’s microstructure was found to be both anisotropic and heterogeneous (Morales-Orcajo et al., 2018), differences in the biomechanical properties (*p_act_, p_pass_, SMC_stretched_*) appear more pronounced depending on the direction of the strip. Higher active and passive stress in the longitudinal direction can be explained by a more longitudinal arrangement of muscle fibers. Locational differences were only observed for *p_pass_* (highest in the trigone). As we found no differences in the contractile behavior of tissue strips from different bladder locations and directions, the detrusor muscle seems to contain a homogeneous and isotropic distribution of predominantly phasic smooth muscle fiber types.

The results of this study will likely contribute to the development and validation of realistic three-dimensional models of the urinary bladder. Bladder models, including biomechanical and structural data, are the base for advancement in the medical understanding and treatment of bladder diseases. For example, bladder augmentation surgery involves an enlargement of the bladder by cutting open the bladder and sewing in a patch of tissue from other organs, such as the intestine or ureter (Greenwell et al., 2001). Bladder functioning will depend on the biomechanical properties of the replacement tissue. Our data will help to develop and prove potential replacement tissues regarding their biomechanical properties. Furthermore, bladder models might be used at the preoperative stage to predict the functional effects of medical operations or treatments (e.g., removal of tumorous tissue, changes in tissue properties due to inflammatory diseases).

## Author Contributions

TS, MaB, and AT conceived and designed the experiments. MiB and AT performed the experiments on biomechanical properties. EM-O performed the histochemical analysis. MiB, AT, NS, and TS analyzed the data, interpreted the results, and drafted the manuscript. MiB prepared the figures. All authors edited, revised, and approved the final version of manuscript.

## Conflict of Interest Statement

The authors declare that the research was conducted in the absence of any commercial or financial relationships that could be construed as a potential conflict of interest.

## Abbreviations

CSA: cross-sectional area
curv: curvature factor
*F_im_*: maximum isometric muscle force
FLR: force-length relationship
FVR: force-velocity relationship
*L*: muscle length
*L*_0_: optimum muscle length associated with *F_im_*
*L_S_*: slack length
*p_act_*: maximum active stress
*p_pass_*: passive stress at *L*_0_
*SMC*_stretched_: lengthwise stretched smooth muscle content
*v_max_*: maximum shortening velocity
*w_asc_*: width of the ascending limb
*w_desc_*: width of the descending limb.
